# Enzymatically oxidized phospholipids restore thrombin generation in coagulation factor deficiencies

**DOI:** 10.1172/jci.insight.98459

**Published:** 2018-03-22

**Authors:** David A. Slatter, Charles L. Percy, Keith Allen-Redpath, Joshua M. Gajsiewicz, Nick J. Brooks, Aled Clayton, Victoria J. Tyrrell, Marcela Rosas, Sarah N. Lauder, Andrew Watson, Maria Dul, Yoel Garcia-Diaz, Maceler Aldrovandi, Meike Heurich, Judith Hall, James H. Morrissey, Sebastien Lacroix-Desmazes, Sandrine Delignat, P. Vincent Jenkins, Peter W. Collins, Valerie B. O’Donnell

**Affiliations:** 1 Systems Immunity Research Institute and Division of Infection and Immunity, Cardiff University, Cardiff, United Kingdom.; 2 Departments of Biological Chemistry and Internal Medicine, University of Michigan Medical School, Ann Arbor, Michigan, USA.; 3 Faculty of Natural Science, Department of Chemistry, Imperial College London, London, United Kingdom.; 4 Institute of Cancer and Genetics, Velindre Cancer Centre, School of Medicine, and; 5 School of Pharmacy and Pharmaceutical Sciences, Cardiff University, Cardiff, United Kingdom.; 6 School of Chemistry, Vanderbilt University, Nashville, Tennessee, USA.; 7 INSERM UMRS 1138, Centre de Recherche des Cordeliers, Paris, France,; 8 Haematology Department, University Hospital of Wales, Cardiff, United Kingdom.

**Keywords:** Hematology, Coagulation

## Abstract

Hemostatic defects are treated using coagulation factors; however, clot formation also requires a procoagulant phospholipid (PL) surface. Here, we show that innate immune cell–derived enzymatically oxidized phospholipids (eoxPL) termed hydroxyeicosatetraenoic acid–phospholipids (HETE-PLs) restore hemostasis in human and murine conditions of pathological bleeding. HETE-PLs abolished blood loss in murine hemophilia A and enhanced coagulation in factor VIII- (FVIII-), FIX-, and FX-deficient human plasma . HETE-PLs were decreased in platelets from patients after cardiopulmonary bypass (CPB). To explore molecular mechanisms, the ability of eoxPL to stimulate individual isolated coagulation factor/cofactor complexes was tested in vitro. Extrinsic tenase (FVIIa/tissue factor [TF]), intrinsic tenase (FVIIIa/FIXa), and prothrombinase (FVa/FXa) all were enhanced by both HETE-PEs and HETE-PCs, suggesting a common mechanism involving the fatty acid moiety. In plasma, 9-, 15-, and 12-HETE-PLs were more effective than 5-, 11-, or 8-HETE-PLs, indicating positional isomer specificity. Coagulation was enhanced at lower lipid/factor ratios, consistent with a more concentrated area for protein binding. Surface plasmon resonance confirmed binding of FII and FX to HETE-PEs. HETE-PEs increased membrane curvature and thickness, but not surface charge or homogeneity, possibly suggesting increased accessibility to cations/factors. In summary, innate immune-derived eoxPL enhance calcium-dependent coagulation factor function, and their potential utility in bleeding disorders is proposed.

## Abstract 2

Innate immune-derived enzymatically oxidized phospholipids enhance calcium-dependent coagulation factor function.

## Introduction

Effective hemostasis requires multiple enzyme/cofactor complexes colocated on an electronegative phospholipid (PL) membrane to first generate thrombin (factor IIa [FIIa]), which then cleaves fibrinogen to fibrin, forming a stable clot. Congenital factor deficiency or acquired hemostatic failure (associated with cardiac surgery) are routinely treated by coagulation factor replacement either individually or as fresh frozen plasma (FFP), while the potential role of procoagulant PLs administered locally has not been considered.

Coagulation factor complexes are tissue factor (TF)/FVIIa (extrinsic tenase), FIXa/FVIIIa (intrinsic tenase), and FXa/FVa (prothrombinase), and they all require PL membranes to function (1–3). TF and PLs are also implicated in infection-associated coagulation (4–6). To support blood clotting, plasma membranes externalize aminoPLs: phosphatidylserine (PS) and phosphatidylethanolamine (PE) (2, 3, 7, 8). PS is required to support coagulation, while PE enhances PS activity(9–11). PS and PE bind coagulation FII, FVII, FIX, and FX in a calcium-dependent manner, through their γ-carboxyglutamic acid (Gla) domains and FVIII and FV through homologous C domains(2, 12–14). Most studies on the interactions of PLs with coagulation factors have focused on head group involvement. However, PLs also differ with respect to the fatty acids (FA) at *sn1* and *sn2,* with saturation and chain length differentially regulating coagulation in vitro (15, 16).

Activated platelets and innate immune cells rapidly generate enzymatically oxidized PLs (eoxPL), including variants of PE and phosphatidylcholine (PC) by the action of lipoxygenases (LOX)(15, 17–24). These lipids, some termed hydroxyeicosatetraenoic acid-PLs (HETE-PL) are localized at or near the membrane surface, with 15-, 12-, and 5-HETE-PLs formed by macrophages/eosinophils, platelets, and neutrophils, respectively. Importantly, HETE-PLs were recently found to be an essential component of normal hemostasis when formed in platelets or eosinophils (25, 26). These observations raised the possibility that eoxPLs could have a procoagulant effect in situations where hemostasis was impaired, and the mechanisms involved now require characterization.

To date, studies of individual coagulation factor interactions with HETE-PLs have not been undertaken, and a full understanding of how they act at a molecular level is lacking. Herein, we investigated their association with factors and their complexes, along with comparing PE and PC analogs and determining which HETE positional isomers best support thrombin generation in plasma. Biophysical parameters of HETE-PL–containing membranes were determined. To investigate their ability to affect hemostasis in situations of impairment, we measured coagulation in plasma deficient in FVIII and in vivo in mice with hemophilia A. EoxPL endogenous levels and their ability to improve thrombin generation in plasma from patients at increased bleeding risk, due to a well-characterized depletion of multiple factors was tested (27). Overall, we found that provision of a procoagulant surface enables coagulation factors to work more effectively in vitro and in vivo, compensating for a relative factor deficiency and improving defective hemostasis. Their ability to promote binding of ions and positively charged proteins relates to improved steric access to PS. In summary, eoxPL facilitated enhanced coagulation factor activities on membrane surfaces. Further studies are required to establish whether these findings represent a potential new therapeutic strategy for acquired and genetic bleeding disorders.

## Results

### HETE-PLs enhance coagulation in FVIII-deficient plasma, acting in part through stimulating intrinsic tenase (FIXa/FVIIIa).

To test the ability of HETE-PLs to support coagulation in FVIII-deficient plasma (the situation with hemophilia A), we used liposomes with the following composition: HETE-PE liposomes were 65% di-stearoyl-PC (DSPC), 30% 1-stearoyl-2-arachidonyl-PE (SAPE), and 5% 1-stearoyl-2-arachidonyl-PS (SAPS), with SAPE replaced with <10% HETE-PE. HETE-PC liposomes were 55% DSPC, 10% 1-stearoly-2-arachidonyl-PC (SAPC), 30% SAPE, and 5% SAPS, with SAPC replaced with <10% HETE-PC. Thus, the only difference between control and HETE-PL liposomes was the HETE hydroxyl group. All were made in the presence of 10 pM recombinant human TF and used at 4 μM lipid concentration.

**Figure 1:**
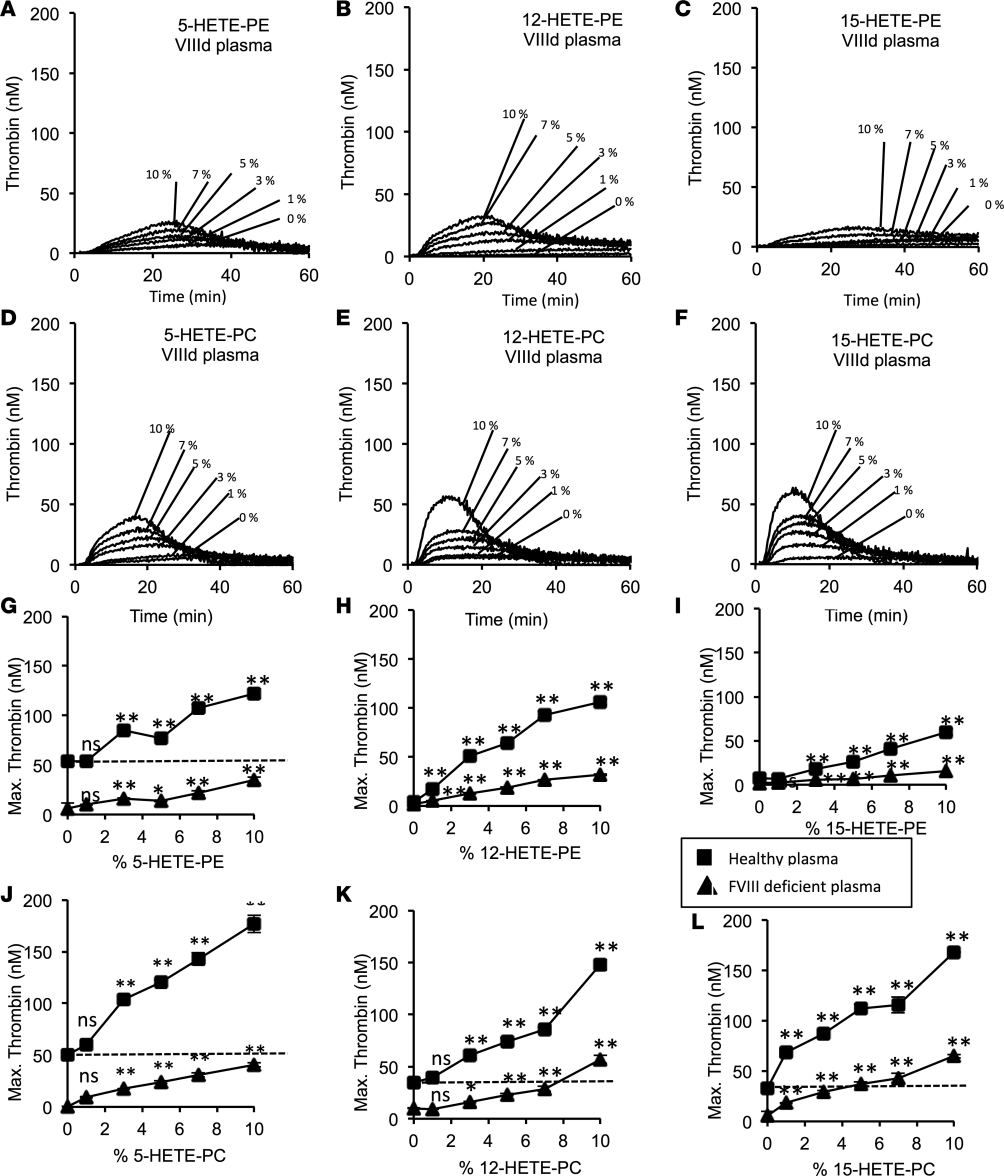
Hydroxyeicosatetraenoic acid–phospholipids (HETE-PLs) restore thrombin generation in FVIII-deficient plasma. Thrombin generation was measured using thrombinoscope as indicated in Methods using either healthy pooled or FVIII-deficient plasma. (**A–F**) Representative traces showing effect of HETE-PL positional isomers on thrombin generation in FVIII-deficient plasma. These were repeated 3 times to generate data shown in **G–L**. (**G–L**) Summary data showing the effect of dose dependence of HETE-PLs on coagulation (*n* = 3, mean ± SEM). Square, normal plasma; triangle, FVIII deficient plasma, **P < 0.01, *P < 0.05 compared with no HETE-PL, as determined by 1-way ANOVA and post hoc Tukey tests.

**Figure 2:**
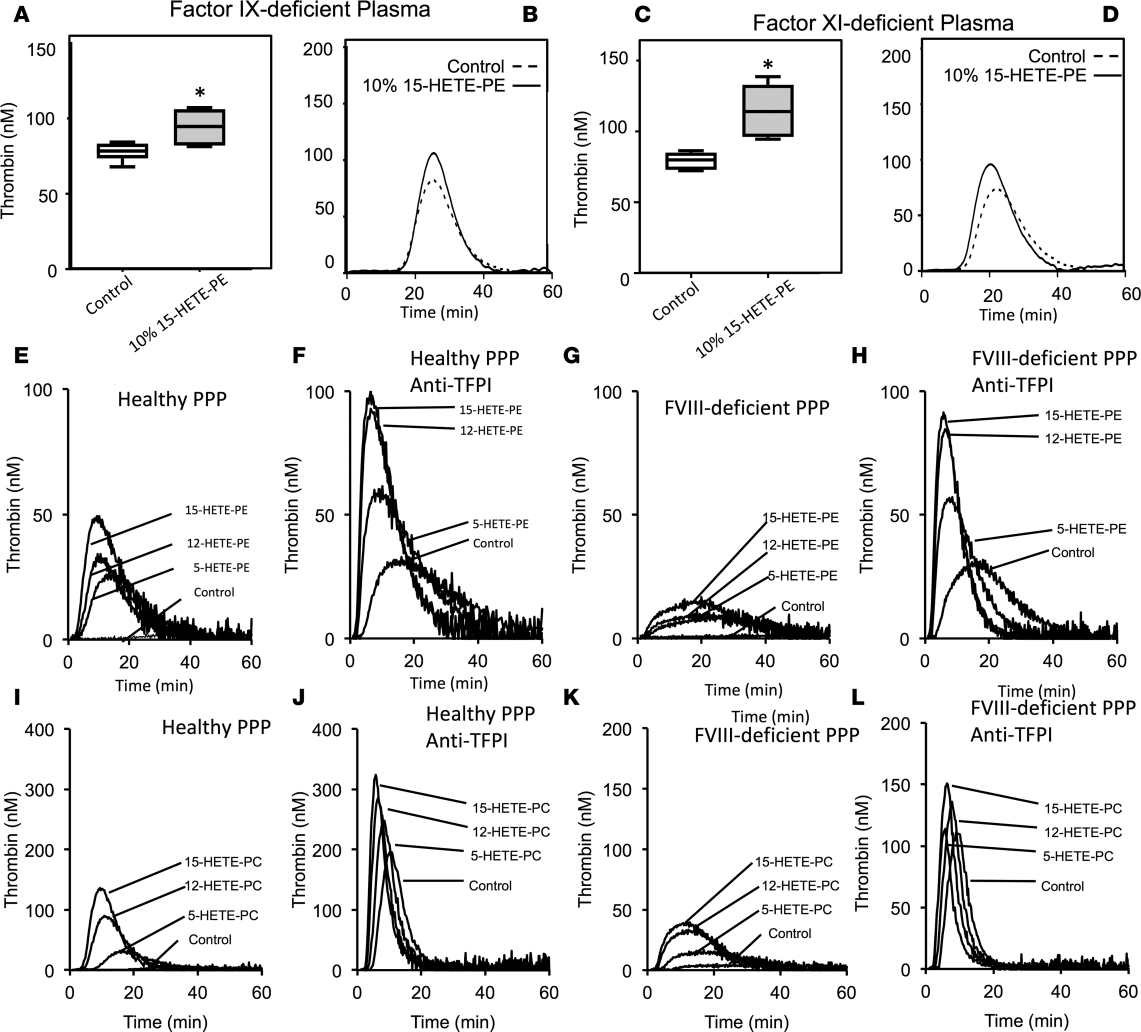
HETE-PLs enhance thrombin generation in the absence of FIX or FXI, or in the absence of tissue factor pathway inhibitor (TFPI) and FVIII combined. (**A** and **B**) Thrombin generation was initiated by addition of liposomes to plasma deficient in FIX, as described in Methods. Liposomes were generated as described in Methods, and 10% 1-stearoyl-2-arachidonyl-PE (SAPE) was replaced with 10% 15-HETE-PE where indicated. (**C** and **D**) Thrombin generation was initiated by addition of liposomes to plasma deficient in FXI, as described in Methods. Liposomes were generated as described in Methods, and 10% SAPE was replaced with 10% 15-HETE-PE where indicated. (**A–D**) *n* = 3, mean ± SEM, *P < 0.05. A representative trace is shown for each. (**E–H**) Thrombin generation was initiated by direct addition of liposomes to plasma as described in Methods, at 10% HETE-PE, and measured in the absence (**E** and **G**) or presence (**F** and **H**) of 100 nmol/l anti-TFPI antibody preincubated for 15 minutes, in either normal (**E** and **F**) of FVIII-deficient plasma (**G** and **H**). (**I–L**) Thrombin generation was initiated by direct addition of liposomes to plasma as described in Methods, at 10% HETE-PC, and measured in the absence (**I** and **K**) or presence (**J** and **L**) of 80 nmol/l anti-TFPI antibody preincubated for 15 minutes, in either normal (**I** and **J**) of FVIII-deficient plasma (**K** and **L**). Data is shown as Tukey box plots, with 1-way ANOVA with Tukey post hoc multicomparison test. *P < 0.05.

In FVIII-deficient plasma, 5-, 12-, or 15-HETE-PL liposomes supported a significant dose-dependent increase in thrombin generation (Figure 1). For 12- or 15-HETE-PLs, thrombin generation was restored to levels that were higher than HETE-PL–free control values obtained using healthy plasma (Figure 1, H, I, K, and L). This demonstrates that the lipids can overcome a clinically relevant factor deficiency to promote hemoastasis. Also, it shows that the procoagulant action of HETE-PLs that we recently demonstrated in healthy plasma is at least partially dependent on FVIII (25). Although HETE-PLs enhanced thrombin generation in FVIII-deficient plasma, they were considerably more active in healthy plasma (seen as steeper slopes in concentration dose–response curves in Figure 1, G–L, for healthy vs. FVIII-deficient plasma). We note that different pooled plasma preparations contain different amounts of coagulation factors and inhibitors; thus, HETE-PE–free controls are not identical between separate experiments. Within single experiments, the same platelet-poor plasma (PPP) preparation was always used for controls and samples containing HETE-PLs. HETE-PL also increases thrombin generation in the absence of FIX (a model for hemophilia B) and FXI (Figure 2, A–D).

### HETE-PL enhancement of thrombin generation is increased by blocking TF pathway inhibitor, indicating that they stimulate extrinsic tenase/prothrombinase activities.

**Figure 3:**
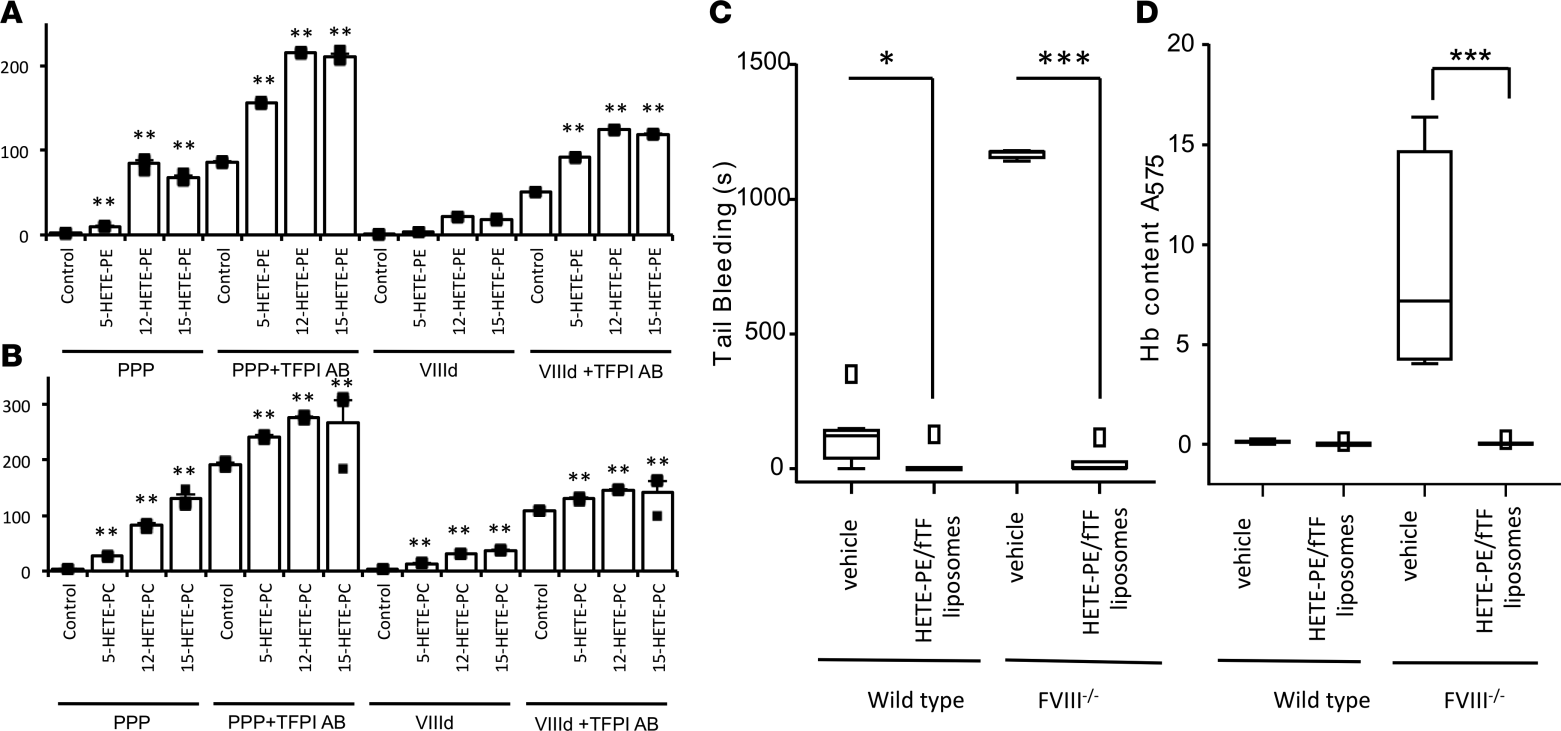
HETE-PL restoration of hemostasis in mice lacking FVIII and enhancement of thrombin generation in FVIII deficiency, with/without tissue factor pathway inhibitor (TFPI) inhibition. (**A** and **B**) Summary data for thrombin generation in plasma as shown in Figure 3, E–L (*n* = 3, mean ± SEM). **P < 0.01, compared with control, as determined by 1-way ANOVA and post hoc Tukey tests. (**C** and **D**) 12-HETE-PE restores hemostasis in mice lacking FVIII. Male mice (11 weeks) were administered 78 ng HETE-PE in liposomes as described in Methods before a 3-mm tail cut. Bleeding time and Hb loss were determined as in Methods (*n* = 4–9 mice per group). Data is shown as Tukey box plots, with 1-way ANOVA with Tukey post hoc multicomparison test. *P < 0.05, ***P < 0.005, data shown as squares in box plot are outliers (values 1.5–3 times lower or higher than the interquartile range).

To determine which factor complexes were stimulated by HETE-PL, we examined the effect of blocking the TF pathway inhibitor (TFPI) using anti-TFPI. This allows TF/FVIIa to work at maximal rates and to directly cleave FX to FXa. In this condition, the activation through the extrinsic tenase (TF/FVIIa) is favored above the intrinsic tenase (FVIIIa/FIXa). In normal and FVIII-deficient PPP, anti-TFPI enhanced thrombin generation in the absence of HETE-PLs, as expected (Figure 2, E–L, and Figure 3, A and B). This is evidenced by control values in each representative trace being consistently higher in the presence of anti-TFPI, and it is consistent with higher activity of TF/FVIIa, leading to direct cleavage of FX to FXa. Inclusion of 5-, 12-, or 15-HETE-PL further increased thrombin generation in this situation (Figure 2, F, H, J, and L). For either healthy or FVIII-deficient plasma, similar thrombin generation rates were seen when TFPI was blocked (Figure 2, F, H, J, and L, and Figure 3, A and B). Notably, in this case, where intrinsic tenase is either lacking (FVIII deficiency) or relatively bypassed (anti-TFPI), thrombin generation enhancement by HETE-PL is equal, indicating that the lipids can act by stimulating extrinsic tenase (TF/FVIIa) and/or prothrombinase (FVa/FXa).

The fold-increase in peak thrombin observed with HETE-PLs in the presence of anti-TFPI was less than in the absence of anti-TFPI and was no longer dependent on FVIII (Figure 3, A and B). This is also consistent with a model in which HETE-PLs stimulate the activities of all coagulation complexes in tandem.

### HETE-PEs prevent bleeding in mice lacking FVIII.

To extend our findings with FVIII-deficient human plasma to an in vivo model, we administered 12-HETE-PE (78 ng)/TF liposomes to FVIII-deficient mice, which are unable to stop bleeding following a tail cut. Strikingly, this resulted in an almost total restoration of hemostasis in the FVIII-deficient mice, without any infusion of exogenous FVIII and with blood loss less than that seen in mice with normal hemostasis treated with vehicle control liposomes (Figure 3, C and D). This provides proof-of-concept that provision of a HETE-PL/TF surface represents a potential topical or locally administered treatment to reduce blood loss in situations where thrombin generation is impaired due to coagulation factor deficiency.

### HETE-PLs stimulate activity of TF/FVIIa, prothrombinase, and tenase complexes.

**Figure 4:**
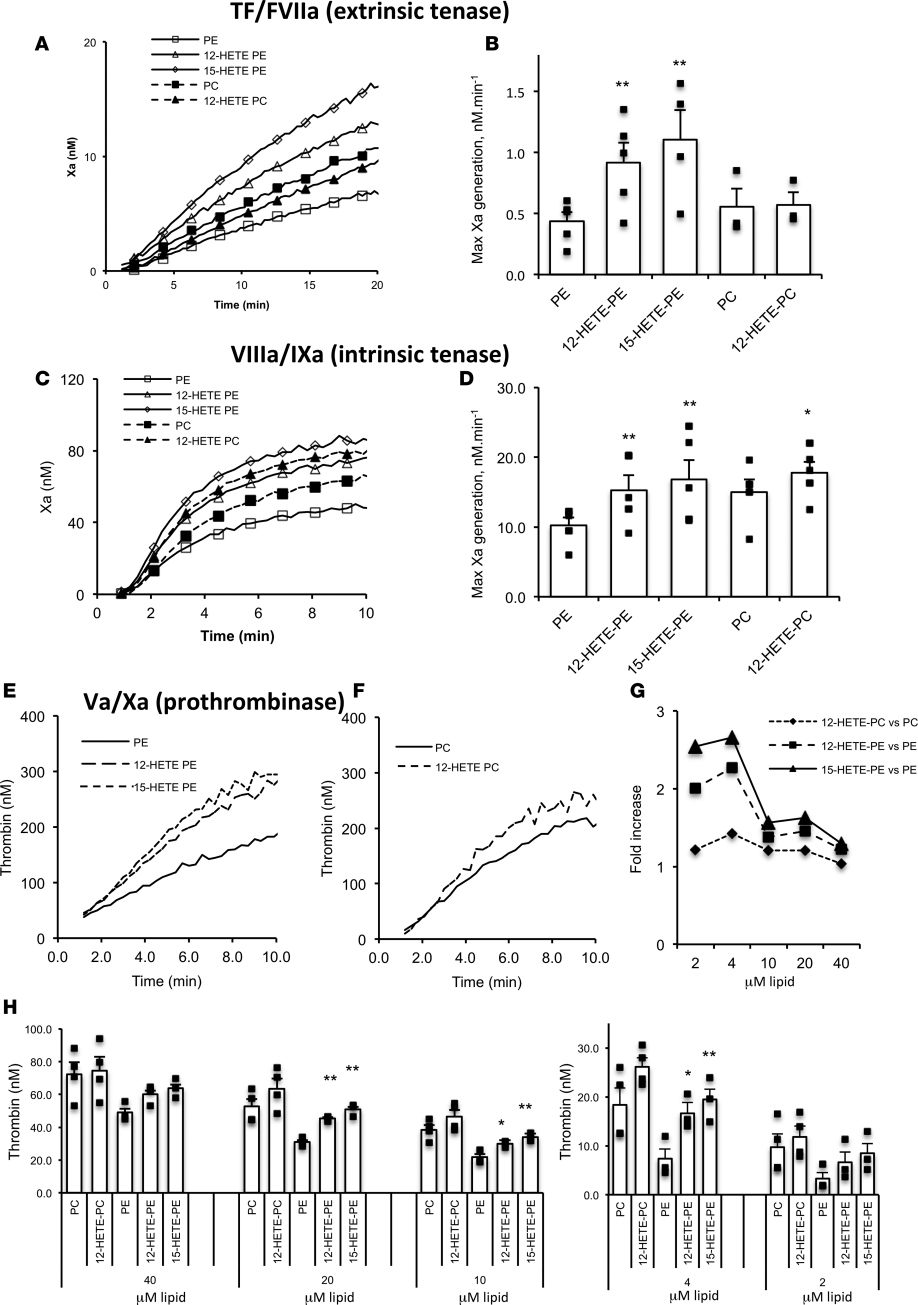
HETE-PLs enhance extrinsic tenase (FVIIa/TF), intrinsic tenase (FVIIIa/FIXa), and prothrombinase (FVa/FXa) activities in vitro. (**A** and **B**) HETE-PLs enhance extrinsic tenase activity. Liposomes (4 mM) containing 50 pmol/l TF were added to FVIIa (10 nmol/l) and FX (136 nmol/l). Liposome composition for PE was 65% DSPC, 5% SAPS, 20% SAPE, with 10% SAPE (control) or 10% HETE-PE; for PC, it was 55% DSPC, 5% SAPS, 30% SAPE, 10% SAPC (control) or 10% HETE-PC. (**A**) Representative traces are shown from 1 of 4 experiments. (**B**) Summary data showing maximal thrombin generation (*n* = 4 separate experiments, each in triplicate, mean ± SEM). (**C** and **D**) HETE-PLs enhance intrinsic tenase activity. FVIIIa (300 pmol/l) was generated using FIIa and then inhibited by hirudin. This was added with substrate to FIXa (80 nmol/l), FX (136 nmol/l), and liposomes (without TF), and FXa generation was measured. (**C**) Representative traces from 1 of 3 experiments. (**D**) Summary data for maximal FXa generation (*n* = 3, mean ± SEM). (**E–H**) HETE-PLs enhance prothrombinase activity. Liposomes (4 mM lipid, composition as above, with no TF) were added to FVa, FXa, and FII at 26 nmol/l, 136 nmol/l, and 1.4 μM respectively. (**E** and **F**) Representative data showing time courses for control and HETE-PL liposomes (*n* = 3 technical replicates, mean ± SEM). (**G**) Fold-changes at different lipid concentrations for HETE-PLs (differences between means, shown in **H**). (**H**) Prothrombinase activity in the presence of liposomes, at different lipid concentrations (*n* = 3–4, mean ± SEM) **P < 0.01, *P < 0.05, as determined by 1-way ANOVA and post hoc Tukey tests.

To further determine the relative sensitivity of individual enzyme/cofactor complexes to HETE-PLs, purified coagulation proteins were tested at physiological concentrations with eoxPL-containing liposomes. Extrinsic tenase (TF/FVIIa) activity was first determined by measuring cleavage of FX to FXa. This was significantly increased by 12-HETE-PE or 15-HETE-PE, but not by 12-HETE-PC (Figure 4, A and B). As control, HETE-PLs had no effect on cleavage of the chromogenic substrate by FXa (data not shown). Similarly, all 3 HETE-PLs enhanced intrinsic tenase (FVIIIa/FIXa), with a lower effect for HETE-PC than -PEs (Figure 4, C and D). Last, 12- and 15-HETE-PE substantially increased thrombin generation by FXa/FVa (prothrombinase), while 12-HETE-PC was ineffective (Figure 4, E and F). Overall, HETE-PEs were more effective than PC analogs, with only HETE-PC significantly elevating intrinsic tenase activity. We note that enhancement of individual complexes is lower than the overall effect on the full cascade, as would be expected due to amplification effects of multiple coagulation pathways working in tandem, as in plasma.

We next determined the effect of HETE-PLs on K_m_^app^ and V_max_ of TF/FVIIa, FVIIIa/FIXa, and FVa/FXa. These experiments use pico- or nanomolar concentrations of enzymes and cofactors much lower than used for assays earlier, undertaken under physiological conditions. Surprisingly, significant differences were not observed using this approach (Supplemental Table 1; supplemental material available online with this article; https://doi.org/10.1172/jci.insight.98459DS1). We hypothesized that this was due to the higher lipid/coagulation factor ratios used here. This was tested by measuring prothrombinase activity, varying lipid concentrations from 2–40 μM. Enhancement of thrombin formation by HETE-PL was greater at lower lipid concentrations (Figure 4, G and H). These data show that HETE-PLs are more effective when PL is limited, most likely by enabling proteins to colocalize in smaller areas at higher concentrations, although further work is required to confirm this conclusion definitively. Limited availability of aminophospholipids is expected to be the physiological situation when platelets are activated during hemostasis.

### HETE-PLs reduce size of liposomes and increase thickness of the bilayer without significantly changing electronegativity, mobility, or size uniformity.

**Figure 5:**
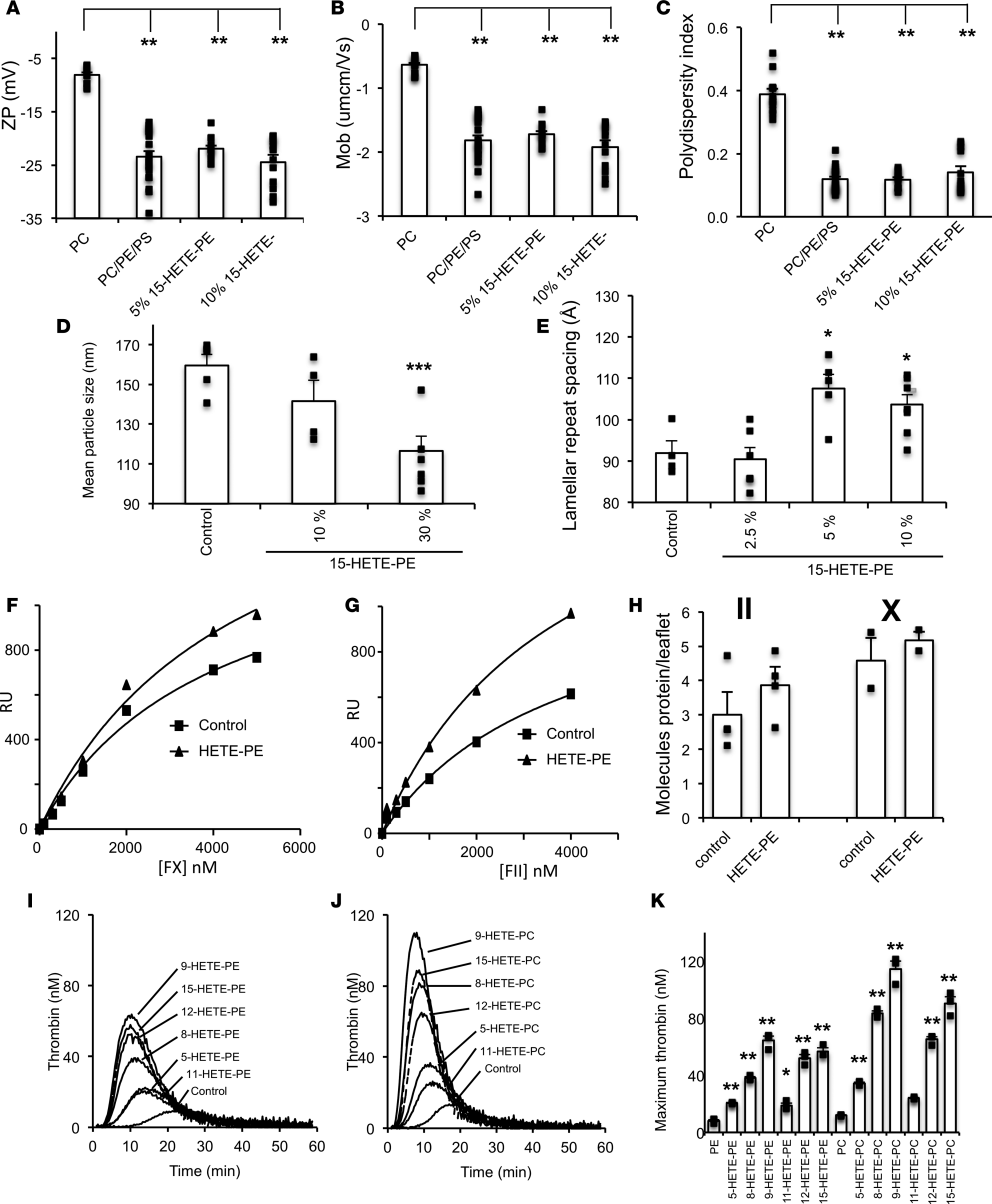
HETE-PLs containing membranes directly bind Gla domain proteins, and specific HETE-PL positional isomers optimally support coagulation. (**A–C**) Liposomes were analyzed using DLS for ζ potential, electrophoretic mobility, and polydispersity. Liposomes (65% DSPC, 5% DSPC, 10%–0% SAPE, 0%–10% 15-HETE-PE) were analyzed using a Zetasizer Nano, as described in Methods (*n* = 3–5 separate liposome preparations, each analyzed as 4 replicates) and compared for differences against PC liposomes, using 1-way ANOVA with multicolumn comparison using Tukey. **P < 0.01. (**D**) Liposomes (65% DSPC, 5% DSPC, 30%–0% SAPE, 0%–0% 15-HETE-PE) were analyzed by nanoparticle tracking analysis to determine the mean diameter (*n* = 4, mean ± SEM). (**E**) X-ray diffraction was carried out at beamline I22 (Diamond Light Source). Samples in glass capillary tubes were held at 37°C ± 0.1°C, and images were integrated as described in Methods (*n* = 3, mean ± SEM). (**F–H**) SPR analysis of protein binding to nanodiscs demonstrates increased binding in the presence of HETE-PE**.** Control discs were composed of 15% SAPS, 30% SAPE, and 55% DSPC. Nanodiscs containing HETE-PE were composed of 15% SAPS, 20% SAPE, 55% DSPC, and 10% HETE-PE. (**F**) Representative SPR-derived binding isotherms for FX binding to HETE-PE and control nanodiscs. Discs used were of the same composition as described for **A**. (**G**) Representative SPR-derived binding isotherms for prothrombin binding to HETE-PE containing and control nanodiscs. Discs used were of the same composition as described **A**. (**H**) Molecules of protein bound per leaflet of nanodiscs in the presence or absence of HETE-PE. Summary data for experiments, *n* = 4 (FX), 2 (FII). (**I–K**) Positional isomer of HETE-PL influences potency of thrombin stimulation. Thrombin generation was initiated by addition of tissue factor containing liposomes to plasma as described in Methods. (**I**) liposomes contained 65% DSPC, 5% SAPS, and 30% SAPE, with 10% SAPE replaced with 10% HETE-PE. (**J**) Liposomes contained 55% DSPC, 5% SAPS, and 30% SAPE, with 10% SAPC replaced with 10% HETE-PC. (**K**) Summary data for maximum thrombin generation (*n* = 3, mean ± SEM). ***P < 0.005, **P < 0.01, *P < 0.05, as determined by 1-way ANOVA and post hoc Tukey tests.

We recently showed that HETE-PLs support enhanced PS-dependent binding of calcium and the positively charged β2GP1 to membrane surfaces, suggesting they may increase electronegativity (25). To characterize the biophysics of these liposomes, ζ potential (analogous to surface charge), electrophoretic mobility, polydispersity index (size uniformity), and diameter of liposomes containing varying 15-HETE-PE amounts were determined. The ζ potential of PC-only liposomes became significantly more negative on incorporation of physiological amounts of native PE and PS, consistent with the hypothesis that aminoPLs enhance electronegativity of cell membranes (Figure 5A). Inclusion of 5% or 10% 15-HETE-PE, in place of SAPE, caused no further increase (Figure 4A). Consistent with this, electrophoretic mobility of the liposomes also decreased with PE/PS incorporation but did not further change with HETE-PE (Figure 5B). Liposome uniformity significantly improved (lower polydispersity) with PE/PS but was not further changed by HETE-PE (Figure 5C). In contrast, HETE-PE led to a significant decrease in liposome size (Figure 5D). Next, lamellar liquid crystalline lipid assemblies were examined to measure bilayer thickness in the presence of 15-HETE-PE. Small angle X-ray diffraction (SAXS) gives repeat spacing of these structures, attributed to changes in bilayer thickness, although we note that it is measured under rather nonphysiological conditions, using lipid mesophases and at room temperatures; thus, it may not be fully representative of a cellular lipid membrane. A significant increase in the lamellar repeat spacing from around 90 Å with 0%–2.5% 15-HETE-PE to around 105 Å with 5%–10% 15-HETE-PE was seen, indicating membrane thickening (Figure 5E).

Overall, the data indicate that the inclusion of aminoPL mediates major increases in electronegativity; however, HETE-PE liposomes are generally smaller (suggesting greater curvature), with a thicker bilayer. The lack of increase in ζ potential indicates that locating the HETE –OH group near the membrane surface, as we previously showed using molecular dynamics simulation, does not formally change surface charge (25). This suggests that HETE-PE may increase accessibility of the phosphate to positively charged proteins/ions, possibly through reduction of steric hindrance, promoting the ability of membrane lipids to form H-bonds with ions/proteins. Whether these biophysical changes are associated with the enhancement of coagulation reactions seen with HETE-PL requires further study.

### Surface plasmon resonance demonstrates increased FII- and FX-binding to HETE-PE.

Nanodiscs represent a potentially novel method for examining protein binding to lipid membranes. Unlike liposomes, they are flat, allowing an assessment of the effect of charge alone, without curvature. Using nanodiscs, the number of FX or FII molecules binding to bilayers containing HETE-PE increased, although the effect was small (Figure 5, F–H). It is anticipated that the cumulative effect of several coagulation factors associating with membranes at higher rates, and in the presence of curvature, would lead to amplification of coagulation in vivo.

### HETE-PL positional isomers vary in their ability to enhance thrombin generation.

Oxidation of arachidonate to form HETEs can occur at 6 positions on the hydrocarbon backbone. To determine whether the position of the –OH group conferred specificity, HETE-PL isomers were tested separately. All isomers enhanced thrombin generation in healthy plasma, with the magnitude varying but the pattern being the same for HETE-PE and PC (Figure 5, I–K). Thus, considering only enzymatically generated isomers, 12- and 15-HETE-PLs generated by activated platelets/murine macrophages and human monocytes/eosinophils, respectively, have the greatest effect on thrombin generation in plasma, with neutrophil 5-HETE-PL being less effective.

### Generation of HETE-PLs is reduced following cardiopulmonary bypass.

**Figure 6:**
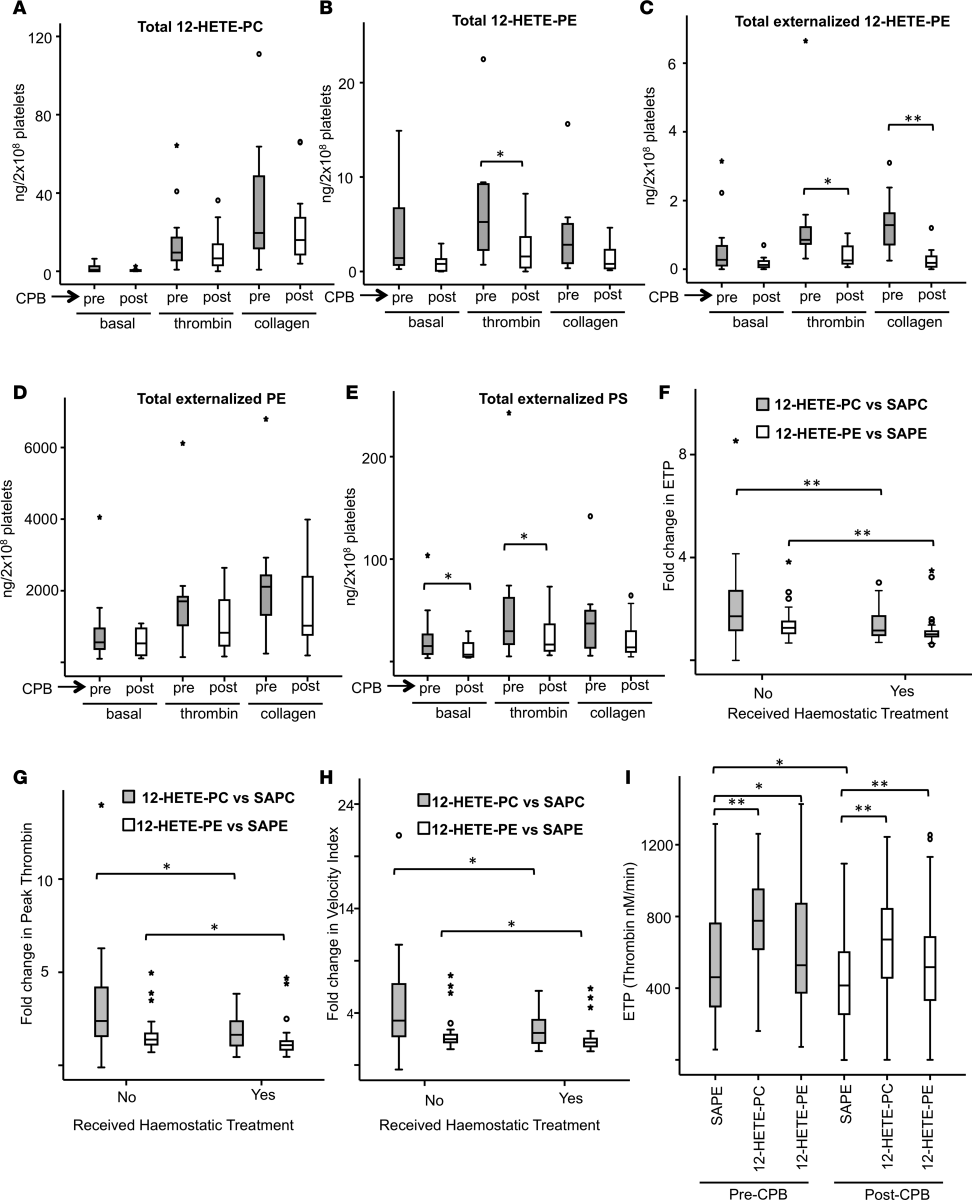
Altered generation and action of HETE-PLs in a clinical setting of hemostatic failure associated with cardiopulmonary bypass surgery. (**A–E**) Platelets after CPB generate less HETE-PE and externalize less HETE-PE and PS than those isolated before CPB. Platelets isolated from peripheral blood of patients, before (shaded) and after (unshaded) CPB were measured for HETE-PE, HETE-PC, and externalized PE, PS, and HETE-PE basally and following thrombin or collagen activation, as described in Methods (*n* = 12). (**F–H**) Stimulation of thrombin generation in preoperative plasma by 12-HETE-PL was significantly reduced in patients who received hemostatic treatment after CPB. Plasma from patients before CPB was added to 50 pmol/l TF liposomes with/without 10% HETE-PL, and thrombin was measured as described in Methods. ETP (**F**), peak thrombin (**G**), and velocity index (**H**) were determined and compared with responses to liposomes that contained native PL, to give fold changes for each. (**I**) Reduced thrombin generation seen in post-CPB plasma was restored using HETE-PL. Thrombin generation was compared in plasma before or after CPB using liposomes containing HETE-PL, as indicated (10%). For **A–I**, horizontal black lines represent median, boxes represent the interquartile range, bars represent values falling within 1.5 times the interquartile range, and asterisks/circles represent outliers (values 1.5–3 times lower or higher than the interquartile range). *n* = 87 for panels **F–I**. Data were analyzed using Wilcoxon rank test for paired variables, Mann Whitney *U* test for unpaired variables and Friedman’s 2-way analysis of variance with post hoc testing to compare repeated measures of related variables, **P < 0.01, *P < 0.05.

We previously showed that HETE-PEs externalize to the outside of platelets following their generation, while HETE-PC is expected to be there already, since it is the predominant external facing PL in these cells (22). To investigate whether generation and externalization of HETE-PLs as well as native PE and PS externalization is altered in a clinical situation associated with elevated bleeding risk and coagulation factor depletion (acquired), platelets were isolated from patients before cardiopulmonary bypass (CPB), at the time of anesthesia, and immediately after CPB, following heparin reversal (the indications for surgery are given in Supplemental Table 2, *n* = 12). Platelets were activated using thrombin (0.2 IU/ml) or collagen (10 μg/ml); then, lipids were extracted and measured for 12-HETE-PLs and externalization of total PE, PS, and 12-HETE-PE using (liquid chromatography-tandem mass spectrometry) LC/MS/MS. After CPB, platelets generated significantly less 12-HETE-PE (in response to thrombin) and externalized less 12-HETE-PE (in response to thrombin or collagen) or native PS (basal or in response to thrombin) (Figure 6, A–E). Trends toward lower levels and/or reduced externalization were evident for all lipids measured after CPB, either basally or after collagen/thrombin stimulation (Figure 6, A–E). These data suggest that, in addition to the recognized platelet storage pool lesion that leads to reduced platelet aggregation (28), depressed activation of platelet LOX and/or scramblase activities may contribute to the elevated bleeding risk observed after CPB through suppressing generation of the procoagulant surface required for effective hemostasis.

### 12-HETE-PL liposomes enhance thrombin generation more effectively in patients who did not require hemostatic treatment after CPB.

Pre- and post-CPB plasma archived from 87 patients was tested for its ability to generate thrombin in the presence/absence of HETE-PLs (Supplemental Table 3). The samples taken after CPB had significantly reduced coagulation factor levels and thrombin generation, as reported (27). Plasma obtained before CPB was used with liposomes containing either native PL (5% SAPS, 30% SAPE, 65% SAPC) or with 10% 12-HETE-PE or 12-HETE-PC in place of SAPE. When measuring endogenous thrombin potential (ETP, total thrombin generated), peak thrombin activity, or velocity index (time to reach maximum thrombin), the fold increase in thrombin generation to HETE-PL liposomes was lower in patients who required FFP for clinically observed hemostatic impairment postoperatively (Figure 6, F–H). These data suggest that patients who develop clinical hemostatic impairment are overall less sensitive to HETE-PLs, even before surgery. As platelets after CPB are also less able to generate a thrombogenic surface (Figure 6), this may further compromise thrombin generation and contribute to excessive bleeding. The mechanistic reasons for the difference in plasma responses to HETE-PLs between the 2 patient groups before CPB remains to be explored.

### HETE-PLs enhance thrombin generation in post-CPB plasma.

Herein, we showed that HETE-PLs can improve thrombin generation in human plasma with FVIII deficiency (Figure 1). Similarly, we examined whether they enhance thrombin generation in post-CPB plasma. As expected, thrombin generation was significantly lower in plasma after CPB (vs. before CPB) (Figure 6I) due to reduced levels of coagulation factors, as previously reported (27). However, thrombin generation was significantly increased by the inclusion of HETE-PLs, although the increase in post-CPB plasma appeared slightly lower. Along with our data using FVIII-deficient plasma and mice, this further supports the idea that eoxPL have the potential to improve coagulation in the context of a bleeding defect due to reduced levels of coagulation factors.

## Discussion

Human platelets, monocytes, neutrophils, and eosinophils acutely generate HETE-PLs via LOXs that remain cell associated, are elevated in thrombotic disease, and were recently shown to enhance hemostasis in WT mice in vivo, although the mechanisms were not fully clarified (17–19, 21, 22, 24, 25, 29). We hypothesized that provision of an enhanced procoagulant surface could represent a therapeutic opportunity in the context of excess bleeding by partially compensating for coagulation factor deficiency. In studies designed to look for proof of principles of this hypothesis, we show that coagulation can be enhanced in FVIII-, FIX-, and FXI-deficient human plasmas in vitro (Figures 1 and 2). In hemophilic mice in vivo, we showed that locally administered HETE-PL/TF liposomes prevented bleeding without the need for added FVIII (Figure 3). Furthermore, HETE-PE/TF liposomes corrected thrombin generation in post-CPB plasma deficient in multiple coagulation factors (Figure 6).

Mechanistically, HETE-PL enhancement of thrombin generation in FVIII-deficient or anti-TFPI–supplemented plasma indicated that HETE-PLs can partially compensate for an absence of FVIII and bypass the hemophilic defect. This may, in part, explain why people with hemophilia have variable bleeding phenotypes and thrombin generation in platelet-rich plasma, because platelets from different individuals will generate variable amounts of HETE-PLs (30, 31). Hemophilic mice without FVIII bled excessively in a standard tail-clip model, as expected. Following local administration of HETE-PE/TF containing liposomes, bleeding was virtually eliminated, despite no FVIII replacement therapy being given. This suggests that, in some situations such as dental extraction or removal of superficial skin lesions, local administration of HETE-PE/TF containing liposomes could reduce the need for FVIII replacement. This hypothesis is untested and requires further study, and experiments in a relevant cutaneous wound model would be interesting (32). The finding that HETE-PLs also enhanced thrombin generation in the absence of FIX or FXI suggests a role in hemophilia B and FXI deficiency. Bleeding after CPB is associated with significant coagulation depletion of multiple coagulation factors (including FVIII), reduced thrombin generation, and an acquired platelet storage pool deficiency, which is known to impair platelet aggregation and adhesion (27, 33, 34). Herein, we showed that post-CPB platelets also had a reduced ability to externalize aminoPLs (native PS and PE) and generated reduced 12-HETE-PLs (Figure 6, F–H). This was associated with a decreased ability for these platelets to generate thrombin and could be an additional mechanism for hemostatic impairment following CPB. Addition of HETE-PL liposomes to plasma from patients obtained after CPB improved coagulation, despite multiple coagulation factor deficiencies, suggesting that these patients could be responsive to lipid-dependent coagulation stimulation strategies. This may explain, in part, why donor platelet transfusions, which can generate normal amounts of HETE-PL, are often useful for arresting bleeding after CPB. Further studies are required to show whether these findings are reproducible, and the clinical implications of the findings are, at present, speculative.

Separately, we found that pre-CPB plasma from patients who subsequently had clinical evidence of hemostatic impairment responded less well to the enhancing effects of HETE-PLs, suggesting an inherent difference in coagulation prior to surgery (Figure 6, F–H). The reasons for this remain unknown and require further investigation.

The procoagulant mechanism of HETE-PLs may involve increased interaction of coagulation factors and calcium at the platelet surface, as recently suggested (25). Herein, we tested which individual factors and enzyme complexes (FVa/FXa, FVIIIa/FIXa, and TF/FVIIa) are sensitive and, furthermore, which positional isomers of HETE-PL are most effective. In isolation, all enzyme complexes were enhanced by HETE-PE; however, only intrinsic tenase was significantly enhanced by HETE-PC (Figure 3). In contrast, we found that, in plasma — where all factors act in concert — coagulation is slightly more sensitive to HETE-PC than -PE (Figure 5, I–K). It is possible that the action of coagulation inhibitors such as antithrombin, protein C, and TFPI in plasma may also be affected by HETE-PLs, but this has not been tested. Overall, our data using recombinant factors or human plasma (FVIII-deficient or with anti-TFPI) support the idea that HETE-PLs act to improve coagulation through stimulating several factor complexes in tandem via a common mechanism, which is independent of the headgroup and entirely dependent on the oxidized FA moiety.

FII, FVII, FIX, and FX contain Gla domains, specialized regions that contain posttranslational modifications of several glutamate residues by vitamin K–dependent carboxylation to form Gla. These mediate a high-affinity interaction of calcium with negatively charged aminoPL on the cell surface, specifically PS and PE (11, 13). The cofactors FVIII and FV bind negatively charged PL via the homologous C1 and C2 domains. Here, we found using nanodiscs that HETE-PLs improved Gla domain protein binding; however, potential additional interactions with cofactor C1/C2 domains may also occur in plasma and have not yet been explored. We note that the enhancement in plasma was greater than when testing recombinant factors, indicating that cooperativity between complexes occurs in plasma to orchestrate full blood clotting.

HETE-PL liposomes have a thicker bilayer and are slightly smaller, indicating greater curvature (Figure 5, D and E). It is known that membrane curvature supports binding of other charged proteins to membranes, including amyloid-β, huntingtin, lactadherin, and Ras (35–38). However, since HETE-PE also enhances factor binding to flat nanodiscs, curvature alone doesn’t explain the effect (Figure 5, F–H). We have previously suggested that the polar –OH group causes the *sn2* acyl chain to push headgroups apart, increasing interactions with phosphate groups (25). We also found that aminoPLs increase surface charge/mobility, but no further elevation was seen with HETE-PE. This indicates that charge-dependent interactions are increased by HETE-PL independently of formally altering electronegativity. This is similar to the action of PE, generally considered to support coagulation through enhancing interactions of PS phosphates with positively charged ions/proteins, rather than through mediating direct factor binding or charge alterations itself. Whether these changes to membrane properties are linked to the enhanced coagulation reactions demonstrated with HETE-PL described herein is not known.

Enhancement of factor activity was greatest at lower lipid concentrations, indicating that the total surface area is a determinant; thus, enhancement by HETE-PLs is greater when available PL surface is limited (Figure 4, G and H). It is possible that a smaller surface area enables greater local concentration of factors, enhancing interactions and activity rates, in concert with increased curvature. In support of this idea, activated platelets were shown to concentrate annexin V and several factors and charged proteins at “ring” structures, suggesting that procoagulant/charged lipids localize in specialized areas at the cell surface — not across the whole platelet membrane (39–42). Whether this phenomenon is linked to the enhancement of coagulation reactions described here requires further investigation.

We found that HETE-PLs differed in their ability to support thrombin generation depending on the position of oxidation. Of the enzymatically generated isomers, 5-HETE-PL — which is generated by neutrophils — had the weakest effect (Figure 5K). The –OH group on 5-HETE is located very close to the acyl carbonyl group and also to the PL phosphate. Thus, it will be already adjacent to the electronegative region of the membrane, without any change in the localization of the FA hydrocarbon chain being required. This is very different to other HETEs, where the –OH will be pushed further down into the nonpolar core when the FA is pointing downward. Thus, the impact of the –OH on the orientation of the FA is likely to be greater for all other HETEs than it would be for this positional isomer. This is a potential explanation for its relatively weaker effect and in line with our idea that the HETEs (except for this one) serve to push the headgroups apart in the membrane. This is, at this stage, a hypothesis.

In summary, we characterize the interactions of endogenously generated eoxPL with individual factor complexes and show proof-of-principle studies for a potential clinical relevance in genetic/acquired bleeding disorders. The findings further our knowledge regarding how physiological membrane oxidation regulates innate immune cell function during acute activation/inflammation.

## Methods

### Materials.

FVIII-, FIX-, and FXI-deficient plasmas were from Helena Biosciences UK. FII, FIIa, (human plasma–derived) FVII, FVIIa, FIX, FIXa, FX, FXa, and hirudin (recombinant),were from Enzyme Research Laboratories. FV and FVa (human plasma–derived), corn trypsin inhibitor (CTI, recombinant), and full-length TF (human recombinant) were from Haematologic Technologies Inc. FVIII (Advate, human full-length recombinant) was from Baxter Healthcare. FX chromogenic substrate S-2765 (N-a-Z-D-Arg-Gly-Arg–para-nitro-aniline [pNA]) was from Quadratech Diagnostics. FIX chromogenic substrate CS-51 (CH3SO2-D-CHG-Gly-Arg-pNA) was from Hyphen Biomed, FII chromogenic substrate Spectrozyme (H-D-HHT-Ala-Arg-pNA) was from Axis-Shield, and the FII fluorogenic substrate (Z-Gly-Gly-Arg-AMC) was from Bachem. Anti–human-TFPI goat polyclonal antibody (catalog AF2974) was from R&D Systems. T-cal Thrombinoscope calibrator was from Stago. SAPE, SAPC, SAPS, and 1,2-DSPC were from Avanti Polar Lipids. MeOAVM (2,2’-Azobis[4-methoxy-2,4 dimethyl-valeronitrile]) was from WAKO. All other chemicals and lipsofast membranes were from MilliporeSigma, except N-methyl Benzohydroamic acid (NMBHA), synthesized in-house according to ref. 43. Solvents were from Thermo Fisher Scientific.

### Synthesis of HETE-PEs/PCs.

HETE PE/PCs were synthesized as previously described, in a high-O_2_ atmosphere (5–12 hours, 37°C) (44). SAPE (or SAPC; 0.2 M) was incubated with 0.3 M NMBHA and 0.02 M MeOAMVN in chlorobenzene. When 50% of the total lipid was oxidized, the lipid hydroperoxides were reduced using 2 moles equivalent of triphenylphosphine, followed by stirring at 20°C for 1 hour. After drying, the sample was redissolved in ~1 ml acetonitrile and loaded onto 2 semi-preparative Discovery C18 columns placed in a tandem sequence (5 mm pore). The sample was eluted isocratically using 5% MeOH in water, separating the 5-, 8-, 9-, 11-, 12-, and 15-HETE-PC/PEs, as detected by their absorbance at 235 nM and individually identified by MS. HETE-PE/PCs were stored at –80°C under inert gas.

### Isolation of plasma.

Blood was taken into 0.32% sodium citrate and 0.59 U/ml CTI, final concentration 20 μg/ml. Platelet rich plasma (PRP) was aspirated after a 10-minute spin at 460 *g* and then double-spun for 10 minutes at 1,730 *g* to produce PPP. Pooled PPP was made from 4 healthy donors.

### Generation of liposomes.

Liposomes were made by extrusion in 20 mmol/l HEPES, 100 mmol/l NaCl, pH 7.35 (buffer A). Where HETE-PE was substituted for unoxidized PE, the liposomes were 5% SAPS, 20%–30% SAPE, 0%–10% HETE-PE, and 65% DSPC (mol%). Where unoxidized PC was substituted for HETE-PC, the liposomes were 5% SAPS, 30% SAPE, 55% DSPC, 0%–10% SAPC, and 0%–10% HETE-PC. Liposomes were made in the presence of 10 pmol/l full-length recombinant TF and used at a final concentration of 4 μM total lipid unless otherwise stated(22).

### Quantification of blood loss from tail-bleeding assays.

C57/BL6 WT (Charles River Laboratories) and FVIII-deficient mice were kept in constant temperature cages (20°C–22°C) and given free access to water and standard chow. Male mice (11 weeks old) were anesthetized using 5% isoflurane and maintained with 2% isoflurane. Where administered, liposomes (10 μl generated as described above, using either [i] 30% SAPE, 65% DSPC, 5% SAPS, and 2.5 nM TF, or [ii] 20% SAPE, 65% DSPC, 5% SAPS, 10% 12-HETE-PE, and 2.5 nM TF) were injected immediately distal of the cut site and immediately before transection (total HETE-PE, 78 ng/dose). The tail was transected 3 mm from the distal end and immediately immersed in 37°C physiological saline. Bleeding was observed as blood loss, and time for stable cessation of blood flow determined, before killing via cervical dislocation. Blood loss was quantified by measuring hemoglobin (Hb) content of the saline as follows: Hb quantitation was achieved via centrifugation of the tube at 250 *g* for 15 minutes, and resuspending RBCs in 5 ml erythrocyte lysis buffer (8.3 g/l NH_4_Cl, 1 g/l KHCO_3_, and 0.037 g/l EDTA in distilled H_2_O). The concentration of Hb was measured as OD 575 nm using a UVIKON 923 double beam UV/VIS spectrophotometer (Bio-Tek Kontron Instruments) and expressed as absorbance units (AU).

### Collection of patient samples.

Patients undergoing heart valve surgery with or without coronary artery bypass grafting and procedures on the aorta were recruited under a protocol approved by the South West Wales Research Ethics Committee (reference 11/WA/0215). Unfractionated heparin was used as anticoagulant to maintain the activated clotting time (ACT) >400 seconds. Protamine at 1 mg per 100 units of heparin was given after CPB prior to removal of the arterial and venous cannulae. Whole blood for preparation of washed platelets or PPP was drawn from a central venous catheter after induction of anesthesia (before CPB) and after CPB following reversal of heparin. Heparin reversal was defined as ACT returning to within 10% of baseline. ACT was measured using a Helena Actalyke MINI II (Helena Laboratories).

### Patient information and inclusion and exclusion criteria for clinical studies.

The median age was 70 years (interquartile range 59–78 years, range 33–88 years). Inclusion criteria included an age of 18 years or older, elective and urgent cardiac surgical procedures involving valve procedures, resternotomy operations on the aorta, and coronary artery bypass surgery combined with another procedure such as value replacement. Patients on warfarin were included after routine treatment to reverse international normalized ratio (INR), along with patients with any abnormality of preoperative coagulation tests or liver impairment. Exclusion criteria were routine coronary artery bypass grafting as the sole procedure, administration of antiplatelet agents other than aspirin within the last 5 days, a platelet count below 120 × 10^9^/l, Hb less than 100 g/l, or emergency procedures. Requirement for hemostatic therapy was defined as transfusion of FFP after the reversal of heparin. FFP was infused if the clinicians managing the patient identified clinical evidence for hemostatic impairment. The laboratory investigators were not aware of whether FFP had been infused until after assays had been completed.

### Isolation of human washed platelets in clinical studies.

Washed platelets were prepared from whole blood drawn from a central venous catheter into syringes containing acidified citrate dextrose (ACD; 85 mM trisodium citrate, 65 mM citric acid, 100 mM glucose) at a ratio of 8.1 parts whole blood to 1.9 parts ACD, as described previously (32) and were resuspended in modified Tyrode’s buffer (134 mM NaCl, 12 mM NaHCO_3_, 2.9 mM KCl, 0.34 mM Na_2_HPO_4_, 1.0 mM MgCl_2_, 10 mM HEPES, 5 mM glucose, pH 7.4) at 2 × 10^8^/ml. Platelets (1.1 ml) were incubated at 37^o^C for 10 minutes with 1 mM CaCl_2_, 0.2 U/ml human thrombin (MilliporeSigma), 10 mg/ml collagen (Mascia-Brunelli), or modified Tyrode’s buffer.

### Externalization of platelet PL in platelets from patients.

Biotinylated standards (DMPE-B, DMPS-B) were generated as described (45). For biotinylation of total PE and PS, an 100-μl platelet sample was added to 20 μl of 20 mM EZ-link NHS-Biotin (Thermo Fisher Scientific) dissolved in dimethyl sulfoxide (MilliporeSigma). EZ-link sulfo-NHS-Biotin dissolved in modified Tyrode’s buffer at a concentration of 5 mg/ml and 602 μl was added to the remainder of the sample for biotinylation of external PE and PS. Both samples were then incubated for 10 minutes at room temperature. Lysine was dissolved in modified Tyrode’s buffer at a concentration of 0.0366 g/ml, and 504 μl was then added to the platelet suspension to quench the reaction for a further 10 minutes. Internal standards were added: 10 ng DMPS-B, DMPE-B, DMPC, and DMPE. PLs were extracted using the method described by Bligh and Dyer (46). These were stored at –80^o^C before LC/MS/MS analysis. MS for biotinylated PE and PS was performed on a Q-Trap 4000 (AB Sciex UK Limited) as described previously (45).

### Lipid extraction and LC/MS/MS of oxidized lipids.

Total HETE-PE/PCs were determined as described previously (47). Lipids were extracted using hexane/isopropanol/acetic acid as described below. 1,2-dimyristoyl-PE or -PC (10 ng) was added to each sample before extraction as an internal standard. Lipids were extracted by adding a solvent mixture (1 M acetic acid, 2-propanol, hexane [2:20:30]) to the sample at a ratio of 2.5 ml solvent mixture to 1 ml sample, vortexing, and then adding 2.5 ml of hexane. Following vortexing and centrifugation (300 *g*, 5 minutes), lipids were recovered in the upper hexane layer. The samples were then reextracted by the addition of an equal volume of hexane followed by further vortexing and centrifugation. The combined hexane layers were then dried under a vacuum and analyzed for HETE-PE using LC/MS/MS. Samples were separated on a Luna 3 μm C18 150 mm × 2 mm column (Phenomenex) gradient of 50%–100% solvent B over 10 minutes, followed by 30 minutes at 100% B (Solvent A: methanol/acetonitrile/water, 1 mM ammonium acetate, 60:20:20. Solvent B: methanol, 1 mM ammonium acetate) with a flow rate of 200 μl/min. Electrospray mass spectra were obtained on a Q-Trap instrument (Applied Biosystems 4000 Q-Trap) operating in the negative mode. Products were analyzed in the multiple reaction monitoring (MRM) mode monitoring transitions from the parent ion to daughter ion of 179.1 (12 HETE [M-H]-) every 200 ms with a collision energy of -45-42V. The AUC for the parent to 179.1 was integrated and normalized to the internal standard. For quantification of these lipids, standard curves were generated with purified standards for each HETE-PE/PC.

### Thrombin generation assays.

Thrombin generation assays in plasma were performed using a Fluoroskan Ascent plate reader (ThermoLabsystems). Cleavage of prothrombin to thrombin was measured using 0.5 mmol/l fluorogenic substrate Z-Gly-Gly-Arg-AMC and thrombin activity compared with thrombin calibrator (Stago). Thrombin generation was calculated from raw fluorescence data (48). In some experiments, plasma congenitally deficient in FVIII, FIX, or FXI was used and/or anti-TFPI antibody was added at 80 nmol/l.

### Activity of individual coagulation complexes.

The prothrombinase complex was assayed on the Fluoroskan Ascent plate reader using FII (1.4 μM), FXa (136 nM), and FVa (26 nM) in buffer B. Liposomes were made as above but without added TF. Since FXa directly cleaved the fluorogenic substrate (Z-Gly-Gly-Arg-AMC) in the absence of FIIa and PL, a FXa (136 nM) control was included. The change in fluorescence caused by FXa alone was subtracted from the fluorescence observed with FII, FXa, and FVa to give a measure of FII cleavage to FIIa. The activity of the TF/FVIIa complex was investigated with liposomes made in the presence of 50 pM full-length recombinant TF. FX (136 nM) and rFVIIa (10 nM) in buffer B was added, and conversion of FX to FXa was measured on the Fluoroskan Ascent plate reader using the fluorogenic substrate (Z-Gly-Gly-Arg-AMC), which was not cleaved by rFVIIa/TF mixtures in the absence of lipid. In place of the thrombin calibrator, FXa (136 nM) was used. The intrinsic tenase complex was investigated using liposomes without TF. FVIII was activated to FVIIIa (final concentration, 300 pM) using thrombin (10 nM for 30 seconds), which was then inhibited by excess hirudin (50 nM). With the calcium and fluorogenic substrate (Z-Gly-Gly-Arg-AMC), the generated FVIIIa was immediately added to liposomes, FIXa (80 nM), and FX (136 nM), and the conversion of FX to FXa was measured.

Coagulation enzyme complexes were also analyzed using kinetic approaches, in the presence of 4 μM liposomes with or without 12- or 15-HETE, with reactions being initiated using 5 mM CaCl_2_. The activity of FIXa/FXa/FVIIa was measured in the presence of variable substrate concentrations, obtaining an apparent K_m_ for the enzyme-substrate complex and turnover number (K_cat_).

Intrinsic tenase (FVIIIa/FIXa) activity was measured at 22°C in In buffer C (buffer A + 5mM CaCl_2_, 0.1% Tween-20, 0.01% BSA) as previously described (9), using 1 nM FIXa and 10 nM FVIIIa using buffer B. FVIIIa was generated in situ as above. The reaction was stopped after 30 seconds, with a 4-fold volume excess of 5 mM EDTA in buffer A.

Prothrombinase (FVa/FXa) activity was assayed at 37°C using 1 nM FXa/FVa and variable amounts of prothrombin (0-2 μM) in buffer D (Buffer A + 5mM CaCL2, 0.01% BSA), stopping the reaction after 3 minutes, with an equal volume of 25 mM EDTA in buffer A.

Extrinsic tenase (TF/FVIIa) complex activity was measured at 37°C using 10 pM recombinant TF (rTF) and 10 nM FVIIa in buffer D. The reaction was initiated by addition of FX (0–0.5 μM) or FIX (0–2 μM) in 5 mM CaCl_2_ and stopped after 5 minutes using an equal volume of 25 mM EDTA in buffer A (FX) or the same buffer using 75% ethylene glycol as solvent (FIX), at a final concentration of 30% ethylene glycol after addition of substrate. Similar experiments were conducted using soluble TF (sTF) instead of full-length rTF.

The FXa, FIXa, and FIIa generated in these assays were measured by the amidolytic conversion of S-2765 (1 hour), CS-51 (3 hour), or Spectrozyme-TH (1 hour), respectively. A Biotek 96-well instrument was used to measure absorbance from cleaved substrates due to pNA (405 nM) release. Standard curves were constructed using known amounts of FXa, FIXa, or FIIa, in order to determine the amount of enzyme activated by each of the complexes. In the case of FIXa, the standard curve was also done in the presence of 30% ethylene glycol.

### Measurement of liposome biophysical parameters.

Liposomes were characterized for diameter, polydispersity index, conductivity, electrophoretic mobility, and charge (ζ potential) using a Zetasizer Nano ZS series ZEN3600 fitted with a 633 nm laser (Malvern Instruments Ltd.). Liposomes (50 μl) containing 15-HETE-PE, generated as above, were added to 950 μl deionized water and placed in a clear plastic ζ cell (DTS 1070, Malvern) and equilibrated for 2 minutes at 25°C prior to measurement. Each was measured at least 4 times. Liposome diameter was also measured using by nanoparticle tracking analysis using a NanoSight LM10 system (Malvern Instruments Ltd.) running NTA-software v2.3 and configured with a temperature controlled, 488nm LM14 laser module and a high-sensitivity camera system (OrcaFlash2.8, Hamamatsu C11440). In brief, 4-mM liposomes were diluted 1,000 times in nano-particle free water (Fresenius Kabi) and analyzed under controlled fluid flow at 37°C, as previously described (49). Data were combined from 4 preparations done on different days, with each analyzed 5 times.

### X-ray diffraction studies.

Lipid mesophases were made up as follows in glass vials: 10% DOPS, 45% DOPC, 35%–45% SAPE, and 0%–10% 15-HETE-PE. Solvent was evaporated under N2 and then placed in a rapid-vac for 2 hours to remove residual solvent. Dried lipid film was scraped off using a pipette tip. Water was added to give 60% (wt/wt) hydration. Lipid mixtures were centrifuged to the bottom of the vial and then stirred again using a pipette tip. Samples were then heat-cycled 10 times. For this, liquid N2 was used to freeze the lipid mixture, followed by thawing at room temperature. SAXS experiments were carried out at beamline I22 (Diamond Light Source). The samples were contained in thin-walled glass capillary tubes and held at 37°C ± 0.1°C. The beamline was configured to deliver 12 keV X-rays, and images were captured using a Dectris Pilatus 2M detector at 2.25 m from the sample position. Diffraction images were integrated using a custom software package developed using the IDL programming language and calibrated against silver behenate, which has a well-defined layer spacing of 58.38 Å (50).

### Nanodisc preparation and surface plasmon resonance (SPR).

Nanodiscs of varying lipid compositions were prepared as described with the following modifications (51). Lipids were mixed in buffer containing 56.3 mM deoxycholate at 43°C so as to facilitate solubility. All steps up to the addition of Bio-beads were performed at 43°C. Prothrombin and FX binding to nanodiscs was evaluated using a Biacore 3000 instrument as described (37). Binding isotherms were plotted using the maximal steady-state binding response units (RU) vs. concentration of protein flowed over the chip, from which K_d_ and maximal protein-binding RU (RU_max_) values were derived using the single-site ligand binding equation. Molecules of protein bound per leaflet were determined using the following equation:

([RU_max_/RU_disc_] × [MW_disc_/MW_protein_])/2,

where RU_disc_ equals the RU change from disc loading, MW_disc_ equals the molecular weight of the Nanodisc, and MW_protein_ equals the protein molecular weight.

### Statistics.

Statistics were analyzed using 1-way ANOVA. Post hoc Tukey tests were used to determine *P* values between individual data groups. The Mann Whitney *U* test was applied to examine differences between unrelated variables, while the Wilcoxon Rank test was used to examine differences between related samples. Analysis of multiple related samples was performed using Friedman’s test. *P* < 0.05 was considered significant.

### Study approval.

All blood donations were approved by the Cardiff University School of Medicine Ethics Committee, were with informed consent (SMREC 12/37, SMREC 12/10), and were in accordance with the Declaration of Helsinki. Patients undergoing heart valve surgery with or without coronary artery bypass grafting and procedures on the aorta were recruited under a protocol approved by the South West Wales Research Ethics Committee (reference 11/WA/0215). Tail-bleeding assays in mice were performed under United Kingdom Home Office License PPL/3150.

## Author contributions

Experiments were conducted by DAS, KAR, MD, MA, MR, CLP, NJB, VJT, AW, SNL, JMG, and MH and designed by DAS, CLP, NJB, AC, MH, JHM, VBOD, PWC, and PVJ. JH provided clinical samples. SD and SLD bred and provided FVIII-deficient mice. YGD generated and provided the lipids used in the studies. DAS, VBOD, and PWC wrote the paper. All authors edited the manuscript.

## Supplementary Material

Supplemental data

## Version 1 03/22/2018

Electronic publication

## Version 2 03/14/2019

The article was updated to reflect the CC-BY license.

## Version 3 03/19/2019

Updated copyright license.
