# Supplementary material for: Enzymatically oxidized phospholipids restore thrombin generation in coagulation factor deficiencies
Source: JCI Insight. 2018 Mar 22;3(6):e98459. doi: 10.1172/jci.insight.98459 (PMC5926910; doi:10.1172/jci.insight.98459)
Supplement: Supplemental data [file jciinsight-3-98459-s007.pdf]

SUPPLEMENTARY DATA

			Control		12-HETE-PE		15-HETE-PE	
**Enzyme**	**Cofactor**	**Substrate**	*Vmax* (s^{-1})	*Km (nM)*	*Vmax* (s^{-1})	*Km (nM)*	*Vmax* (s^{-1})	*Km (nM)*
1nM Xa	1nM Va	0-8mM II	0.96 ± 0.04	64.1 ± 7.8	0.77 ± 0.11	75.3 ± 6.0	0.98 ± 0.10	80.1 ± 7.8
1nM IXa	10nM VIIIa	0-500nM X	0.56 ± 0.08	36.3 ± 4.4	0.59 ± 0.09	40.13 ± 5.3	0.51 ± 0.08	37.8 ± 4.1
10pM VIIa	10pM rTF	0-500nM X	2.61 ± 0.23	23.8 ± 4.0	3.49 ± 0.51	28.5 ± 4.9	3.47 ± 0.39	21.3 ± 5.2
10pM VIIa	10pM rTF	0-2mM IX	1.69 ± 0.12	62.1 ± 2.2	1.80 ± 0.08	66.6 ± 3.1	2.06 ± 0.13	72.0 ± 8.1

Supplementary Table 1. HETE-PEs do not enhance VIIa/TF, intrinsic tenase (VIIIa/IXa), or prothrombinase (VaXa) activities using standard kinetic assays. Liposomes were made with/without tissue factor as described in Methods, replacing 10 % SAPE with 10 % 12- or 15-HETE-PE, final lipid concentration 4 μM. V_{max} is expressed as the number of substrate (X or IX, X, II respectively) factor proteins activated per second for each enzyme, where turnover is about 1-3 per second (n = 4-7 per condition, mean ± SEM).

Type of Operation	Number
AVR	3
AVR + another procedure	5
MV Repair/Replacement	2
Redo AVR	2

Supplementary Table 2. Types of operation performed on patients from whom platelet PL were analyzed pre- and post-CPB (AVR = aortic valve replacement, MV = mitral valve). The median time on bypass was 125 minutes (interquartile range 107-170, range 75 - 389 minutes) and median aortic cross clamp time was 99 minutes (interquartile range 84-136, range 48-249).

Type of Operation	Number
AVR	23
AVR + another procedure	36
MV Repair/Replacement	6
MV Repair/Replacement + another procedure	12
Redo AVR +/- another procedure	4
Replacement of ascending aorta +/- another procedure	5
LV aneurysmectomy and CABG	1

Supplementary Table 3. Types of operation performed on CPB patients. (AVR = aortic valve replacement, MV = mitral valve, LV = left ventricle, CABG = coronary artery bypass graft). The median age was 69 (interquartile range 66-77, range 28-88), median time on bypass 144 minutes (interquartile range 111-178, range 57-427), an median aortic cross-clamp time 114 (interquartile range 86-141, range 49-334)
